# Electrotransfer of IL-15/IL-15Rα complex for the treatment of established melanoma

**DOI:** 10.1186/2051-1426-3-S2-P244

**Published:** 2015-11-04

**Authors:** Shawna Shirley, Cathryn Lundberg, Niculina Burcus, Richard Heller

**Affiliations:** 1 OncoSec Medical Inc., San Diego, CA, USA; 2 Frank Reidy Research Center for Bioelectrics, Norfolk, VA, USA; 3 Old Dominion University, Norfolk, VA, USA

## Background

Interleukin-15 (IL-15) mediates the function of some innate and adaptive immune cells, which makes it an attractive target as an anti-cancer agent. Gene electrotransfer (GET) has been used to safely deliver plasmid DNA (pDNA) to tissues in a number of pre-clinical and clinical studies for cancer therapy. Previously we have shown using GET to deliver interleukin-15 (IL-15) to mouse melanoma resulted in long term-tumor regression and survival of a percentage of treated animals after challenge. Here we aim to improve this protection by modulating the levels of IL-15 expressed and stabilizing the protein by co-expressing its soluble receptor IL-15Rα.

## Methods

GET was used to deliver plasmids encoding IL-15 and IL-15Rα, separately as well as on a single plasmid, to established B16.F10 tumors in a three delivery regimen on days 0, 4 and 7. In order to generate therapeutic levels of gene expression, two delivery protocols were utilized. The treated animals were monitored for changes to tumor volume over a period of 9 weeks. Tumor-free mice were challenged with subcutaneous injection of B16.F10 cells on the opposite flank and monitored for an additional 50 days. Over the course of the experiment, samples were collected for histology, IL-15 expression and cytokine analysis.

## Results

The amount of intratumoral IL-15 expressed depends on the plasmid and electrotransfer protocol used. Electrotransfer of IL-15 induces tumor regression in treated animals compared to controls. The presence of IL-15Rα did not significantly enhance these effects. IL-15 EP also promotes long-term survival of treated animals compared to non-GET controls. Mice treated with IL-15/IL-15Rα GET expressing moderate levels of the complex showed greater levels of protection from challenge than other groups that expressed higher levels of the complex and control animals. These mice also had a greater amount of tumor infiltrating CD3e+ cells. High levels of IL-15/IL-15Rα induced high levels of tumor infiltrating CD11+ cells and anti-tumor cytokines.

## Conclusions

IL-15 GET was able to induce tumor regression and increased survival in treated animals, but only those expressing moderate levels of IL-15/IL-15Ra showed systemic effects able to protect the greatest percentage of animals from challenge. The level of intratumoral complex expressed after gene electrotransfer is an important determinant of the therapeutic outcome. This finding will help refine this type of immunotherapy and move it closer to the clinic.

